# Intrinsic molecular vibration and rigorous vibrational assignment of benzene by first-principles molecular dynamics

**DOI:** 10.1038/s41598-020-74872-6

**Published:** 2020-10-21

**Authors:** Shaoqing Wang

**Affiliations:** Shenyang National Laboratory for Materials Science, Institute of Metal Research, Chinese Academy of Sciences, Shenyang, 110016 China

**Keywords:** Optical spectroscopy, Carbohydrate chemistry

## Abstract

Vibrational assignment, which establishes the correspondence between vibrational modes and spectral frequencies, is a key step in any spectroscopic study. Due to the lack of experimental technique to directly observe the thermal vibration of atoms, the assignment is usually done by empirical trial-and-error method with considerable uncertainty. Here we demonstrate a successful study of intrinsic molecular vibration property based on first-principles molecular dynamics trajectory. A unified approach for calculating and assigning vibrational frequencies is developed and applied to solve some historical issues of benzene vibration. As a major achievement, the experimental frequencies of benzene *a*_*2g*_ and *b*_*2u*_ vibrations are reassigned, which breaks a deadlock in contemporary spectroscopic science and removes a cloud over the application of density-functional theory in organic chemistry. This work paves the way for the comprehensive realization of the first-principles spectroscopic research, and provides crucial clues to solve the century-old problems of Kekule resonance, π-deformation, and aromaticity.

## Introduction

Similar to the importance of intrinsic electronic and elastic properties of materials, it is essential to master the intrinsic molecular vibration property in order to understand the structure, various physical and chemical properties of the molecule and to explain the relevant experimental phenomena. However, the intrinsic vibrational behavior of molecules is not directly detected by any existing experimental method and can only be studied indirectly through measuring the spectral information from the excited photons. Molecular spectroscopy is one of the most important research tools in contemporary chemical and physical societies. The key factors concerning the vibration property of molecule are the vibrational modes and the characteristic or fundamental frequency corresponding to each mode. Because there is no any experimental method available to directly observe how the atoms move at a specific frequency, the explanation of experimental molecular vibrational spectrum is strongly dependent on the theoretical vibrational modes analysis^1–3^. Traditional vibrational mode assignment for a specific frequency is usually done with the help of the empirical force field method which solves the problem backward from the available experimental frequencies^1,4,5^. Through calculating and subsequent diagonalizing the force constant matrix, the normal-mode frequencies are directly related to the eigenvalue and the normal modes to the eigenvectors of this matrix. A major task in classical spectroscopy study is to develop suitable force fields for the vibrational mode analysis and frequency calculation^6–8^. Spectral measurement is restricted by the selection rules in quantum transition which results the available frequency information by experiment is limited^9^. The insufficient experimental data may lead to considerable uncertainty in vibrational assignment. On the other hand, the power of first-principles molecular dynamics (FPMD) simulation in accurate calculation of molecular vibrational frequencies through naturally taking anharmonic effect into consideration has been demonstrated^3^.

Benzene, as a famous textbook canonical paradigm, attracts continuous research interests since 1865. It is no exaggeration to say that many important breakthroughs in contemporary chemistry are originated from the study of benzene-related issues. Vibrational mode describes how the atoms in molecule move at a specific frequency. The information is crucial for generating accurate molecular force field. Benzene has thirty vibrational modes and ten of them are double degenerated^10^ which leads to twenty independent vibrational modes and fundamental frequencies. Benzene has five modes that are inactive for any kind of spectroscopic experiment^12^, and therefore cannot be measured directly. Due to the lack of enough experimental support, there are still many controversies about the exact motion pattern of atoms for several vibrational modes of benzene^11^. In the aspect of theoretical research, many reports on the quantum chemistry calculation of benzene vibrational spectrum have been published since the end of last century^13–21^, but the consistency between these theoretical results and the related experimental data is far from satisfactory^13,15,18,20,22–25^. In particular, the calculation of the benzene *b*_*2u*_ vibrational frequencies is unanimously recognized as a very headache problem for theoretical researchers, because the results obtained using almost all of the most advanced quantum chemistry methods in such calculation have encountered serious errors compared with the experimental results^18,26^. This problem is often blamed for a failure to take anharmonic factors into account or insufficient electronic correlation description^23^ in the density-functional theory (DFT) calculations, but is there a deeper reason behind? As a result, the vibration property of benzene has not been fully understood until today. This situation also poses a serious obstacle to the understanding of many crucial properties of benzene and other aromatic compounds, such as the localization or delocalization preference of π-bond^27^, the Kekule resonance^28,29^, the origin and nature of aromaticity^30–32^, etc. All of these are among the key fundamental problems in the contemporary chemistry, especially the organic chemistry.

Here, we demonstrate the first mathematically rigorous study of the full-set fundamental frequency calculation and vibrational mode assignment of benzene at the state-of-the-art first-principles level. On the basis of the raw atomic trajectory through the first-principle molecular dynamics (FPMD) simulation close to zero Kelvin, the Eckart frame algorithm and discrete fast Fourier transform (FFT) are used for noise reduction and spectrogram calculation^3^. The unambiguous assignment for each of the calculated fundamental frequency is subsequently carried out by the frequency-domain filtering algorithm. The strategy in this work enables us to study the intrinsic molecular vibrational behavior at the vibrational ground-state with no need to take photon emission and absorption into consideration. Remarkably, we show that the experimental frequency in the vicinity of 1300 cm^−1^, previously assigned to vibrational mode *ν*_*14*_ in Wilson notation^12^, actually belongs to *ν*_*3*_ in *a*_*2g*_ symmetry, which results in the experimental frequency reassignment of the *ν*_*3*_ and *ν*_*14*_ modes of benzene. The relative errors of the new assigned experimental *ν*_*14*_ frequency is less than 1.0% to our calculated value, and less than 3.0% to the most rigorous DFT calculated results reported up to date, which eliminates the long-standing doubts about the applicability of advanced DFT theory in the study of molecular vibrational problems related to π-bond. The attempt in the present work opens a pathway towards the full realization of first-principles spectroscopic research. These results are of fundamental importance for the final solution to the century-old problems of Kekule resonance, π-deformation, and aromaticity.

## Results and discussion

### Fast Fourier transform algorithm for vibrational assignment

Fast Fourier transform (FFT) is a highly efficient mathematical tool for spectral analysis of discrete time-domain signals. Molecular dynamics simulation produces trajectories of atomic positions as a function of time. FFT converts the time-dependent dynamic evolution of a molecular system in time-domain into spectral domain. Inverse FFT (IFFT) converts a frequency-domain function back to a time-domain function. If only a specific frequency band is allowed in IFFT operation, the resulted time-domain function reflects the vibrational behavior of the molecule at that frequency. An arbitrary atom in the molecule is taken to illustrate the principle of the frequency-domain filtering IFFT algorithm. A time-domain coordinate function, $\mathop{r}\limits^{\rightharpoonup} _{i} \left( t \right) = x_{i}^{1} \left( t \right)\mathop{i}\limits^{\rightharpoonup} + x_{i}^{2} \left( t \right)\mathop{j}\limits^{\rightharpoonup} + x_{i}^{3} \left( t \right)\mathop{k}\limits^{\rightharpoonup}$ (*i* = 0 … *N* *−* *1, t* = i*Δt), is generated through MD simulation with timestep *Δt* and total *N* steps. The vibrational spectrogram of the atom is calculated by the discrete FFT algorithm:

1 $$f^{j} \left( \nu {\left( k \right)} \right) = \mathop \sum \limits_{n = 0}^{N - 1} x_{n}^{j} \left( {t\left( n \right)} \right)e^{{ - 2\pi i\frac{kn}{N}}} , \, \left( {j = 1 \ldots 3} \right).$$

The time-domain trajectory of the atom at a specific frequency band *ν*_*s*_ is obtained by using the IFFT algorithm:

2 $$y^{j} \left( \nu {t\left( n \right)} \right) = \frac{1}{N}\mathop \sum \limits_{k = 0}^{N - 1} f^{j} \left( \nu {\left( k \right)} \right)g\left( \nu {\left( k \right)} \right)e^{{2\pi i\frac{kn}{N}}} , \, \left( {j = 1 \ldots 3} \right).$$

*t(n)* = n*Δt, *g*(*ν*(*k*)) is the frequency-domain filtering function, defined by

3 $$g\left( \nu {\left( k \right)} \right) = \left\{ {\begin{array}{*{20}l} {k_{s} ,} \hfill & {\left| \nu {\left( k \right) - \nu _{s} } \right| \le \Delta } \hfill \\ {1,} \hfill & \nu {\left( k \right) = 0} \hfill \\ {0,} \hfill & {else.} \hfill \\ \end{array} } \right.$$

**Figure 1:**
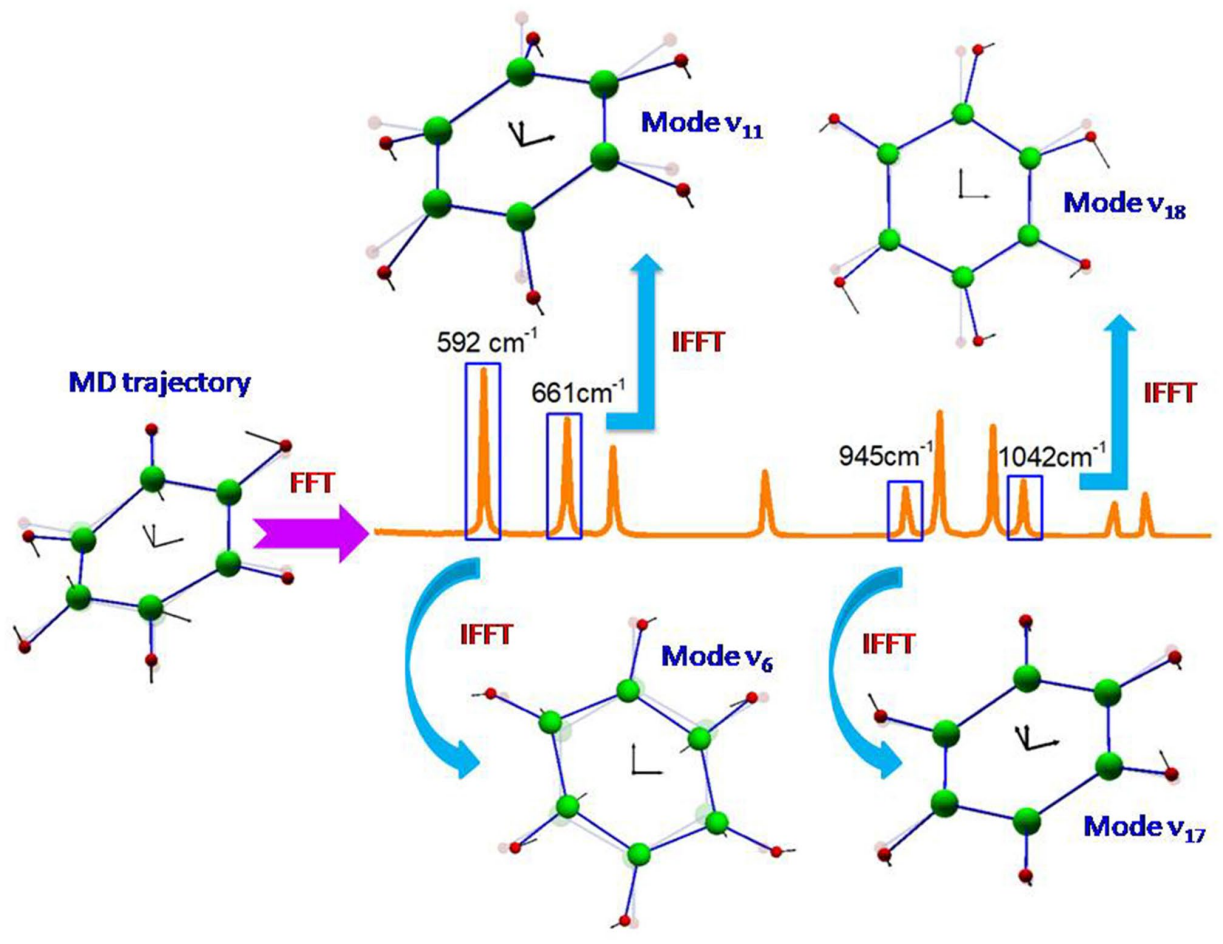
Schematic illustration of Fourier transform algorithm for vibrational assignment of benzene. Spectrogram is calculated through fast Fourier transform of the noise-reduced atomic trajectory by Eckart frame algorithm. Mathematically rigor vibrational assignment is carried out through analyzing the atomic trajectory at the specific frequency band obtained by frequency-domain filtering IFFT calculation. The real and virtual diagrams are the instant and equilibrium configurations, respectively.

The value of *Δ* should ensure that only the specified band can pass through the filtering window. The information of atomic equilibrium position in molecular vibration is given by $f_{k}^{j} \left( 0 \right)$. *k*_*s*_ ≥ 1.0, this parameter is used to amplify the amplitude of the filtered vibrational signal for easy visual inspection because the amplitude of vibrational signal is very small at extremely low temperature^3^. In the current study, the values of this parameter range from 1000.0 of the strongest vibrational mode *ν*_*16*_ to 200,000.0 of the weakest mode *ν*_*14*_. Through analyzing the frequency-specified atomic trajectory generated by the frequency-domain filtering IFFT algorithm, the mathematically rigorous molecular vibrational assignment for each fundamental frequency can be realized. The schematic illustration of the above strategy implementation is shown in Fig. 1.

**Figure 2:**
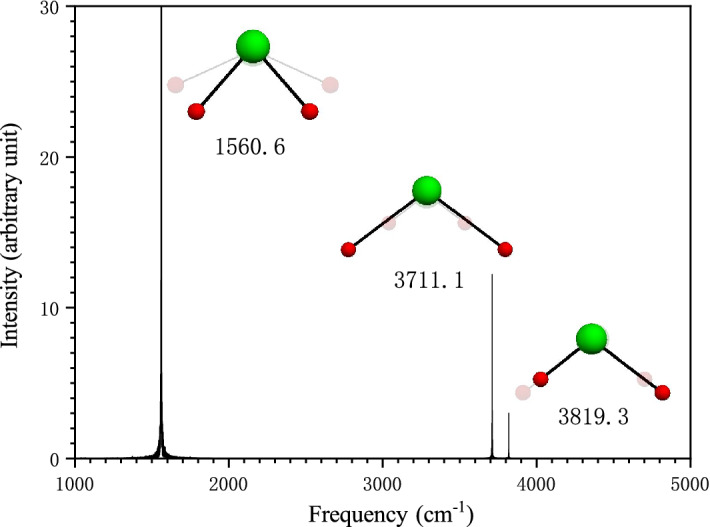
Vibrational assignment of water molecule. The spectrogram was calculated by Eckart frame algorithm in Ref. 3. The inset graphs close to each frequency band were generated by the frequency-domain filtering algorithm, which show the two extreme configurations (the real and virtual diagrams) of water in a vibrational period at the frequency band.

In order to demonstrate the validity and accuracy of the method, the vibrational assignment of water monomer is shown Fig. 2. All the three fundamental vibrational modes of water are infrared active and had been definitely determined by experimental measurements. The relative errors of our calculated fundamental frequencies of water by the Eckart frame algorithm are between − 2.1 and 1.7% compared with the experimental results^3^. Fig. 2 shows that water monomer vibrates in scissor bending, symmetric stretching, and asymmetric stretching at 1560.6 cm^−1^, 3711.1 cm^−1^, 3819.3 cm^−1^, respectively. Therefore, the vibrational modes of these bands are unambiguously assigned to the bending mode of *a*_*1*_ symmetry, the symmetric stretching mode of *a*_*1*_ symmetry, and the asymmetric stretching mode of *b*_*1*_ symmetry in sequence. The animation movies of these modes can be found in the Supplementary MOV files named by the corresponding frequency plus “H2O”.

### Theoretical calculation of the full set of benzene fundamental frequencies

**Figure 3:**
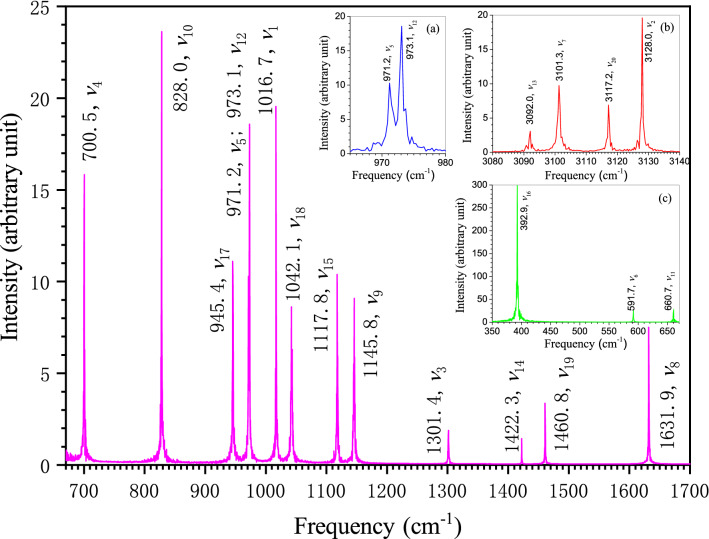
FFT spectrogram of benzene molecule using Eckart frame algorithm. The frequency resolution of the calculated spectrogram is 0.3 cm^−1^. The symbol after the frequency value of each band indicates the assigned vibrational mode by the frequency-domain filtering IFFT algorithm.

Figure 3 shows the FFT spectrogram of benzene molecule calculated by the Eckart frame algorithm^3^. Our calculation uses an atomic trajectory of 2^20^ FPMD steps, which is obtained by simulating an isolated benzene molecule in a cubic box with the edge length of 20 Å. The average temperature of the simulation system is kept at 0.16 K. According to the quantum chemistry, the molecule should only exhibit the intrinsic behavior of its vibrational ground state at such a low temperature. The corresponding vibrational frequencies are only the fundamental frequencies in vibrational ground state. As can be seen from Fig. 3, there are twenty different bands in our FFT spectrogram, which correspond exactly to the twenty fundamental frequencies of benzene. The two bands in the vicinity of 970 cm^−1^ are very close together, but they can still be clearly distinguished in the enlarged illustration of inset (a) in Fig. 3. Despite that the difference in intensity between the strongest and the weakest bands is more than two hundred times, the intensity even for the weakest band has about seventy times stronger than the uniform background noise. This fact confirms the uniqueness of these twenty bands as the fundamental bands of benzene. A general comparison of the frequency distribution between our calculated and the experimental data shows a pretty good consistency in ranging from the lowest frequency around 400 cm^−1^ to the highest frequency around 3100 cm^−1^ (see Supplementary Table 1).

The experimental study of molecular vibrational spectrum is generally restricted by the selection rules of quantum transitions. Although the experimental research on the molecular vibration of benzene has been going on for more than a century, among its 20 fundamental frequencies, there are still several fundamental frequencies that cannot be directly measured through experiment. The relevant experimental data are only indirectly estimated by means of the combinational or rovibrational band analyses^12^. Ingold et al.at the University College of London are the first to carry out a systematic study on the vibrational spectrum of benzene^33,34^. They successfully measured all seven Raman-activated and four infrared-activated fundamental frequencies and estimated the other nine fundamental frequencies through combination band and Teller–Redlich product analyses. The main differences between all subsequent experimental reports^12,35,36^ and their results mainly focus on the three in-plane C-H bending or rocking vibrations in *a*_*2g*_ and *b*_*2u*_ symmetries. The remaining fundamental frequencies that could not be directly measured at that time are still not available by experiment until today, and the data available are still estimates^11,37^. In view of this situation, the current work demonstrates the unique advantage of our FPMD approach in computing the full set of molecular fundamental frequencies.

### Rigorous vibrational assignment of benzene by frequency-domain filtering algorithm

**Figure 4:**
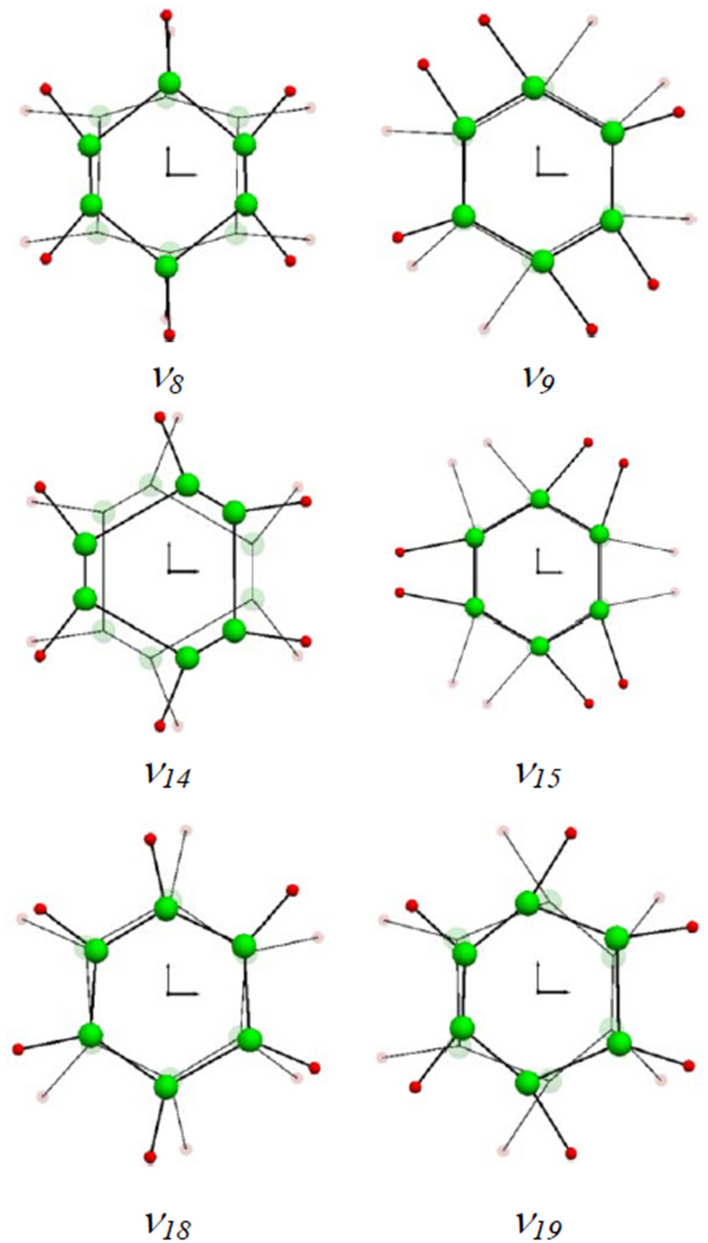
Dual-extreme diagrams of normal mode: *ν*_*8*_ and *ν*_*9*_*, ν*_*14*_ and *ν*_*15*_, *ν*_*18*_ and *ν*_*19*_. The real and virtual diagrams are respectively the molecular configurations at the two extremes of a molecular geometric variable in a vibration period.

**Figure 5:**
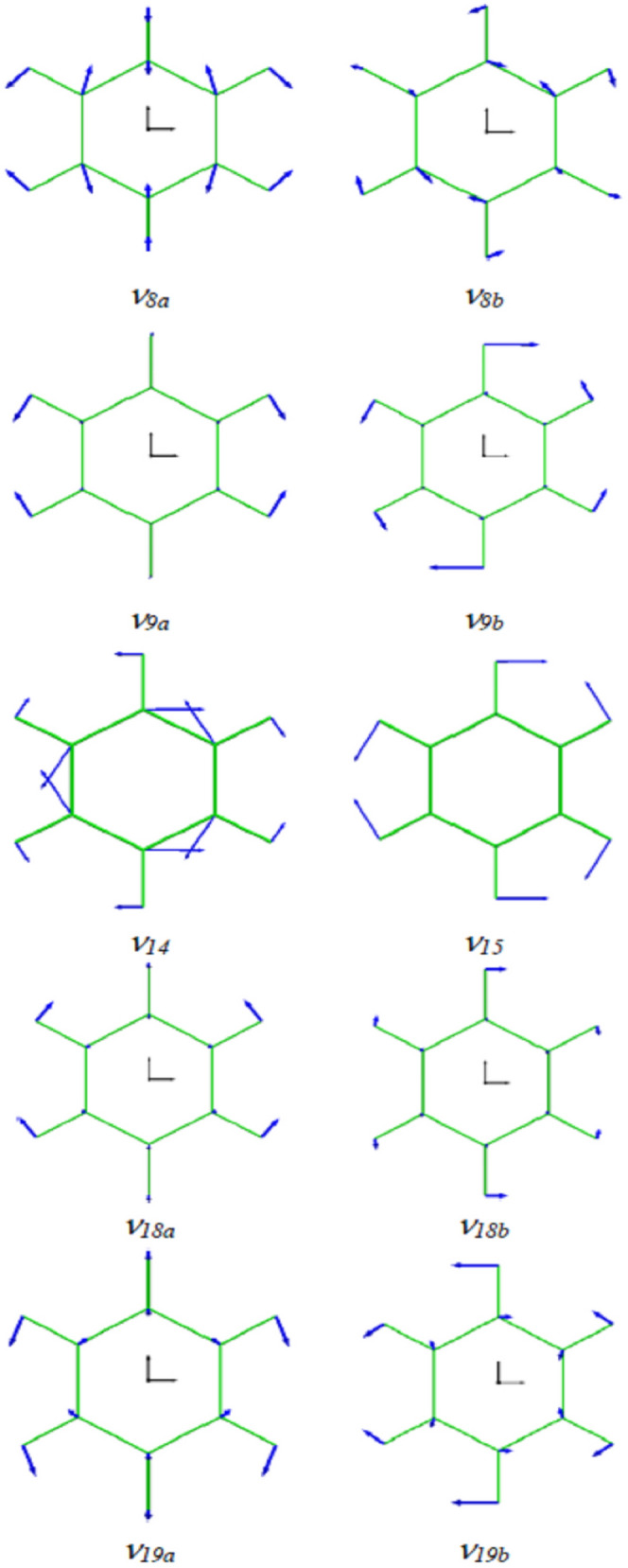
Calculated normal coordinate relative displacements of the six vibrational modes in Fig. 4. The ratios of carbon and hydrogen displacements are 2.105 and 0.038 for *ν*_*14*_ and *ν*_*15*_, respectively. The diagrams for the rest modes are available in Supplementary Fig. 3. The modes with subscript ‘*a*’ or ‘*b*’ are in double degeneracy.

Vibrational assignment relates the spectral frequencies to the vibrational modes. On the basis of the noise-reduced FPMD atomic trajectory by Eckart frame method^3^, the frequency-specified atomic trajectory for each of the twenty fundamental frequencies of benzene in Fig. 3 is generated using the frequency-domain filtering IFFT algorithm. The animation movies for these frequency-specified atomic trajectories are available in the Supplementary MOV files named by the corresponding frequency and mode. With the help of these frequency-specified trajectories, the vibrational mode at a specific fundamental frequency can be unequivocally determined by analyzing the synergic motion of atoms in the molecule within a vibrational period. Wilson normal mode numbering system^10^ is adopted throughout this article. The vibrational mode can be graphically represented by the normal coordinate relative displacement diagram^10^ or by the dual-extreme diagram. The dual-extreme diagram shows the molecular configurations at the two extremes of a molecular geometric variable in a vibrational period (see Supplementary Fig. 1). Such diagram can fully reflect the relative displacement between adjacent atoms in vibration. Our assigned mode for each of benzene twenty fundamental frequencies is given after the corresponding frequency value in Fig. 3. The dual-extreme diagrams of the twenty normal modes are given in Supplementary Fig. 2. The figure shows that the point group symmetries of these vibrational modes are completely in accordance with the Wilson description^10^, except that the atomic displacement patterns of several modes are somewhat different(*ν*_*8*_*, ν*_*14*_*, ν*_*15*_*, ν*_*19*_*, ν*_*20*_). Actually, only partial displacement patterns were given for the ten degenerated modes (*ν*_*6*_ to *ν*_*10*_, and *ν*_*16*_ to *ν*_*20*_), and the displacement pattern of *ν*_*20*_ is obviously incorrect in Wilson original paper. Compared with the other representative results^11,35,38–40^, it is found that our atomic displacement patterns are almost identical to that described by Pitzer and Scott^35^, except that they completely ignored the slight movement of the carbon atoms in *ν*_*2*_*, ν*_*9*_*, ν*_*13*_*, ν*_*15*_ and *ν*_*20*_. Our displacement patterns for the eleven Raman or IR active modes also agree well with the descriptions by Angus et al^33^. The atoms may act as active or passive role depending on their displacement patterns in molecular vibration. The motion of passive atom is driven by active atom. Through examining Supplementary Fig. 2 and the Supplementary Movies, the carbon atoms are found to be the active atoms in *ν*_*8*_, *ν*_*14*_ and *ν*_*19*_ while hydrogen atoms are the active atoms in *ν*_*9*_, *ν*_*15*_ and *ν*_*18*_ which is just in agreement with the conclusion by Herzfeld et al^34^. The adjacent hydrogen and carbon atoms in benzene may move in the same direction or in the opposite direction during vibration. The significant differences on the atomic relative displacement between our study and those reported by Langseth and Lord^40^, Varsanyi^39^, and Page et al^38^. are found in the following six modes in pairs: *ν*_*8*_ and *ν*_*9*_*, ν*_*14*_ and *ν*_*15*_, *ν*_*18*_ and *ν*_*19*_. As seen in Fig. 4, the adjacent hydrogen and carbon atoms move oppositely in *ν*_*8*_, *ν*_*14*_ and *ν*_*19*_ while they move in the same direction in *ν*_*9*_, *ν*_*15*_ and *ν*_*18*_, which is basically consistent with the result by Pitzer and Scott^35^ but essentially the exact opposite of what by Varsanyi^39^ and Page et al^38^. This problem was also noticed recently by Gardner and Wright. Therefore, they suggested that the corresponding frequency (wavenumber) orders of these three pairs of modes should be reversed for keeping the order in the displacement pattern diagrams^11^. However, this change in the frequency order rather than the order of displacement pattern diagram will result in incompatibility with the frequency order reported by almost all experiments to date^12,34–36,41^. By using the information of atomic displacement relative to its equilibrium position provided by the frequency-specified atomic trajectory, the coordinate relative displacement diagrams of these six normal modes are drawn in Fig. 5 (see Supplementary Fig. 3 for the rest modes).

Since the vibrational frequencies of degenerate modes are identical, the frequency-domain filtering algorithm is incapable to generate the frequency-specified vibrational trajectories for each of the modes in degeneracy in principle. The trajectory obtained at the degenerate frequency reflects the overall motion of these in-degeneracy modes. Nevertheless, if the vibrational phases of these modes are different, one can still qualitatively determine the atomic displacement patterns for each of them through analyzing the frequency-specified vibrational trajectory just as shown in Fig. 5. As an alternative solution, it is also possible to completely eliminate the degeneracy of these in-degeneracy vibrational modes by simulating the molecular vibration under the condition of external electric field, but the calculated spectrogram will deviate from the intrinsic property.

The comparison of our frequency calculation and vibrational assignments with some representative published experimental and theoretical results can be found in Supplementary Table 1. Because the classical force field method by experimental data fitting involves a lot of artificial factors, the theoretical calculation data used for the comparison in this table are mainly selected from the strict quantum chemistry calculations in recent years. It can be seen that our results are quite consistent with the reports in these theoretical and experimental publications. However, a very noteworthy issue in this table is that, unlike the consistency of other modes from beginning to end, there are significant differences between early and late experimental assignments of *ν*_*3*_ and *ν*_*14*_. Neither of the two modes is active in the IR and Raman spectra of gaseous benzene. The frequency assigned to *ν*_*3*_ was lower than that assigned to *ν*_*14*_ in all early experimental studies^33–35,40^. The turning point came in 1949, when Mair and Hornig carried out infrared spectroscopic experiments on liquid and crystalline benzene. They found a weak band at 1310 cm^−1^ in their IR spectrum of liquid benzene. With the decrease of temperature, the band becomes sharper and sharper in crystalline benzene, showing the typical characteristics of fundamental band. Therefore, they assigned it to *ν*_*14*_ according to the more relaxed selection rule of condensed state to gaseous state, and reassigned 1340 cm^−1^ to *ν*_*3*_ through combination band analysis^42^. Our frequency assignment of these two modes is in the same order as those early researches. The estimated reasonable frequency ranges of *ν*_*3*_ and *ν*_*14*_ are in 1150–1350 cm^−1^ and 1400–1900 cm^−1^ respectively by Pitzer and Scott^35^, and Herzfeld et al^34^. Our calculated frequencies of the two modes, 1301.4 cm^−1^ and 1422.3 cm^−1^, are fairly within these ranges. Although Mair and Hornig’s *ν*_*14*_ frequency assignment of 1310 cm^−1^ has been widely accepted, it has become a great puzzling problem for the theoretical researchers because no advanced quantum chemistry method has realized its rigorous calculation so far (see Supplementary Table 1). The band at 1301.4 cm^−1^ in our calculated spectrogram is closest to Mair and Hornig’s *ν*_*14*_ assignment with an error only -0.6%. However, our analysis of frequency-specified atomic trajectory has undoubtedly confirmed that the band actually belongs to *ν*_*3*_, in which benzene vibrates in *a*_*2g*_ symmetry. Our *ν*_*3*_ assignment is in line with the early studies and the NIST recommendation^34,35,39^. Supplementary Table 1 shows that the relative error between most of our calculated and the accepted experimental fundamental frequencies of benzene is generally within ±3.0%. However, *ν*_*14*_ is the only exception, with a maximum error of 8.6%.

**Table 1:** vibrational reassignment and band combination analyses of *ν*_*3*_ and *ν*_*14*_ for the experimental IR bands of crystalline benzene, measured at 103 K (− 170 °C)^42^. The data in parentheses is absolute error.

Frequency (cm^−1^)	Original assignment	Reassignment
1312	*ν*_14_	*ν*_3_
1416	*ν*_16_ + *ν*_5_, split	*ν*_14_
2347	*ν*_19_ + *ν*_10_ (5)	*ν*_3_ + *ν*_18_ (− 1)
2907	*ν*_14_ + *ν*_8_ (1)	*ν*_3_ + *ν*_8_ (− 1)
1829	*ν*_17_ + *ν*_10_ (0)	*ν*_14_ + *ν*_16_ (− 2)
2588	*ν*_12_ + *ν*_8_ (− 9)	*ν*_14_ + *ν*_9_ (− 2)

### Experimental infrared spectrum reassignment of crystalline benzene

The understanding of benzene is of fundamental importance in any discussion of aromatic compounds. Benzene holds unusual structural stability in regular planar hexagonal configuration. The phenomenon was initially explained by the Kekule resonance that benzene has a dynamic structure which is in a rapid interchange between its two complementary carbon ring configurations in *b*_*2u*_ symmetry^43^. The modern molecular orbital theory generally considers that it is the delocalized π-bonds formed by the six carbon unhybrid *p*_*z*_ orbitals contributing to the extraordinary stability of benzene^44^. But, this explanation was challenged by the π-deformation theory which states that π-electrons have inherent distortive propensity, it is the energetic preferences of the σ bonds forcing benzene in the regular hexagonal configuration^45,46^. The key basis of the π-deformation theory^7,47^ happens to be the very low frequency of the mode *ν*_*14*_, suggested by Mair and Hornig^42^, in benzene *b*_*2u*_ carbon-ring vibration (Kekule vibration). The *ν*_*14*_, like *ν*_*3*_ and *ν*_*15*_, is closely associated with the anharmonic in-plane C-H wagging vibration, and is key important for the generation of force fields of benzene. However, as mentioned earlier, this mode not only has the problem of inconsistent results in previous experimental researches, but also in theoretical researches. The problem that makes researchers deeply confused is that the *ν*_*14*_ frequency assigned by Mair and Hornig has never been reproduced by rigorous quantum chemistry calculation. As shown in Supplementary Table 1, the frequency of *ν*_*14*_ calculated by our FPMD simulation is very close to that of many of the most advanced first-principles calculations, including by the correlation corrected vibrational self-consistent field (cc-VSCF) theory^48^, Slater–Vosko–Wilk–Nusair (SVWN) functional^18^, and the second-order Moller–Plesset^49^ perturbation theory, but it is more than 100 cm^−1^ higher than the experimental value of Mair and Hornig. Our calculated frequency in the vicinity of 1300 cm^−1^ is closest to Mair and Hornig’s result, but it has been unequivocally assigned to *ν*_*3*_ through our vibrational mode analysis. We noted that there had been several reports of experimental studies on *b*_*2u*_ vibration by the two-photon (TP) fluorescence spectroscopy^50,51^. Because the effective information provided by TP experiment is very limited, the interpretation of the relevant experimental data strongly depends on the molecular force field and the vibrational mode determined based on the infrared and Raman experiments^26,51^. Therefore, the fundamental frequency determined by TP technique can only be a slight adjustment to the results by the last two methods. In view of this situation, we raised the doubt that Mair and Hornig might have incorrectly assigned the frequency of *ν*_*14*_.

At the beginning of the last century, Halford^52^ and Horing^5^ respectively suggested that many of the vibrational modes, that are inactive to the Raman or IR spectroscopy in gaseous state, might be activated in liquid or crystalline state with only a slight change in frequency compared to the gaseous state. Based on this judgment, the researchers had conducted extensive experimental measurements of the Raman and infrared spectra of solid and liquid benzene^42,53–56^. Mair and Hornig measured the IR spectra of liquid benzene at 301 K, and solid benzene at three temperatures of 261 K, 208 K and 103 K. They observed a weak but very sharp absorption peak in the vicinity of 1300 cm^−1^, and assigned the band to *ν*_*14*_ for the first-time^42^. However, this frequency is more than 300 cm^−1^ lower than that estimated in the early experiments^34,35^ and nearly 200 cm^−1^ lower than the lowest value calculated in the early theory. Although the acceptance process was rather slow and accompanied by many doubts^39,57–59^, their assignment of *ν*_*14*_ has been followed in most of the subsequent studies.

**Figure 6:**
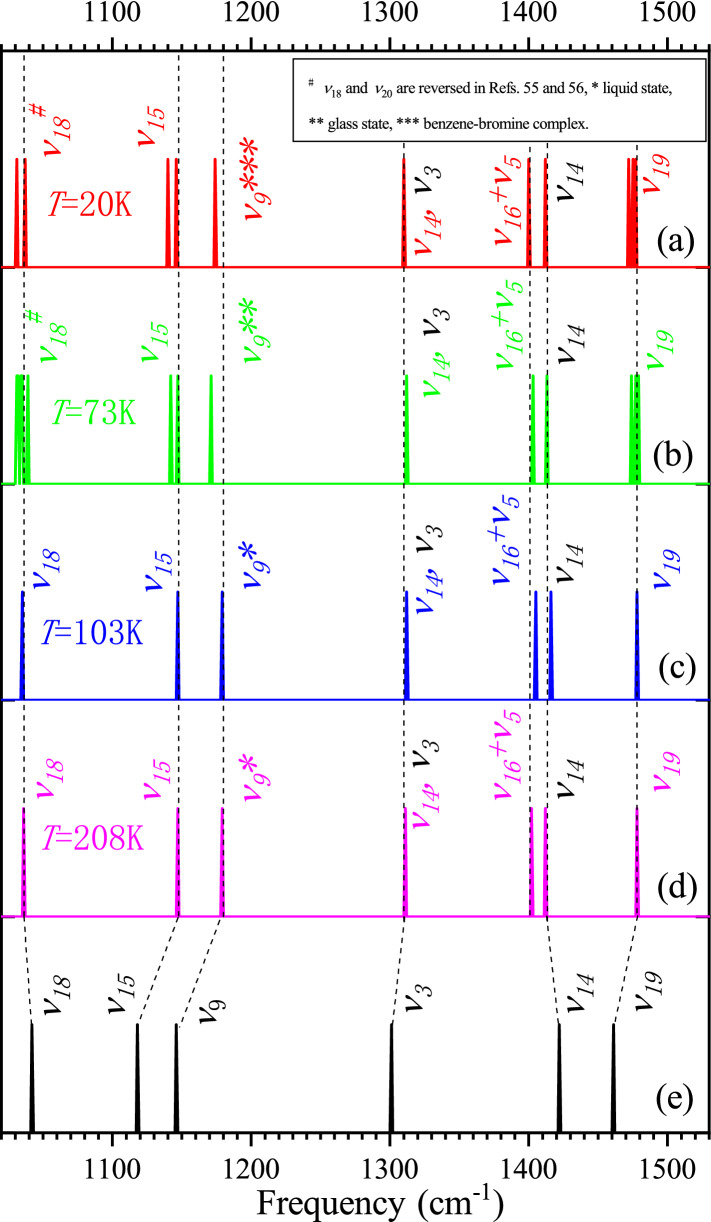
Comparison and vibrational reassignment of experimental IR bands of solid benzene. For the sake of frequency comparison, only the positions of the bands are accurately plotted, regardless of their intensity difference. The frequencies of crystalline benzene measured at temperatures of 20 K^56^, 73 K^55^, 103 K and 208 K^42^ are drawn in (**a**) to (**d**), respectively; the FFT spectrogram of benzene molecule calculated in this work is presented in (**e**). Since mode *ν*_9_ is inactive in crystalline benzene, its frequency was measured by benzene-bromine complex in (**a**), glass benzene in (**b**) and liquid benzene in (**c**) and (**d**), respectively. The modes labeled with the same color as the band line are the original assignments. Our mode reassignments of the experimental frequencies are labeled in black.

**Figure 7:**
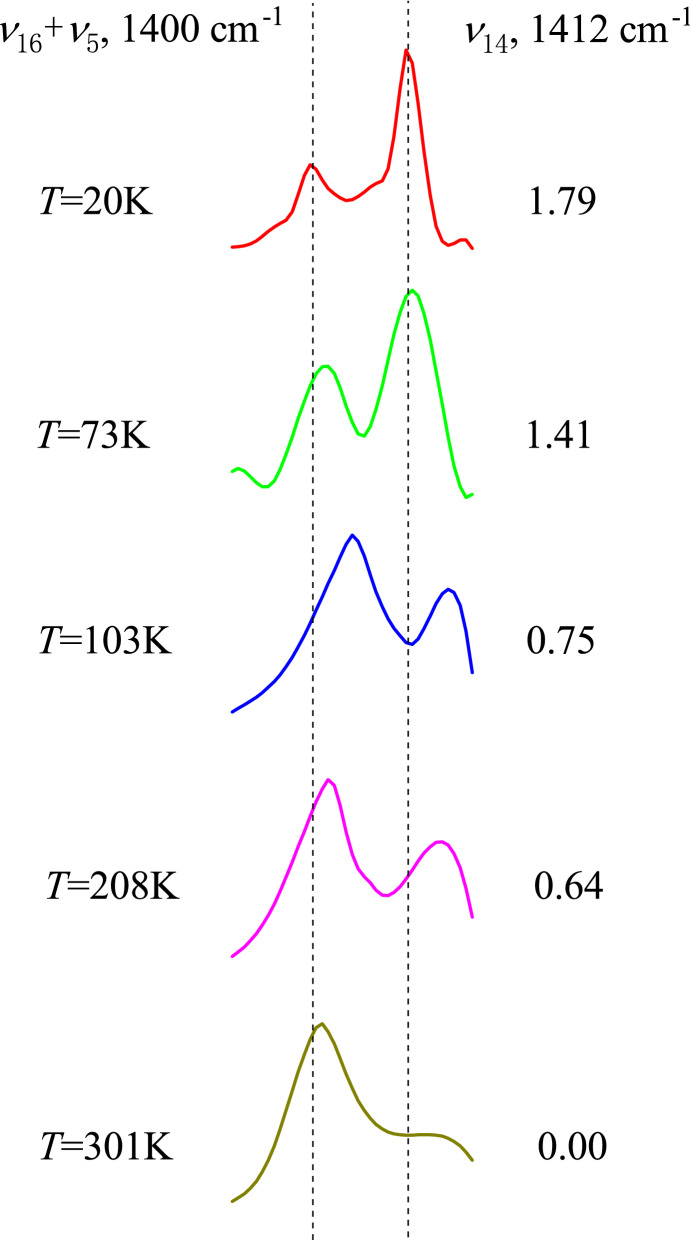
Temperature dependence of the IR absorption two-band structure in the vicinity of 1400 cm^−1^. The figure is drawn based on the experimental data by Mair & Hornig (103 K, 208 K, and 301 K)^42^, Person et al.(73 K)^55^, and Szczepaniak & Person (20 K)^56^. The experimental temperature and peak intensity ratio are indicated in the left column and the right column, respectively. The average spacing between the two bands is 11 cm^−1^. There is a slight frequency shift between different measurements. The first band at 1400 cm^−1^ is assigned to *ν*_*16*_ + *ν*_*5*_^42,55,56^. The second absorption band in the vicinity of 1412 cm^−1^ increases dramatically with the decrease in temperature, confirming that it is the *ν*_14_ fundamental.

Figure 6 compares our vibrational assignment with four representative infrared experimental studies of crystalline benzene^42,55,56^. The frequency range is between 1020 cm^−1^ and 1530 cm^−1^. For the sake of frequency comparison, only the positions of these bands are accurately plotted, regardless of their intensity differences. The experimental results at liquid hydrogen and liquid nitrogen temperatures are given in Figs. 6 (a) and (b), respectively. The Mair and Hornig’s measurements at 103 K and 208 K are drawn in Figs. 6 (c) and (d). The result in Fig. 6 (e) is from this study. It is seen that our vibrational assignment is in accordance with these experimental results except *ν*_*3*_ and *ν*_*14*_. Our study confirms that the band in the vicinity of 1300 cm^−1^ is indeed a fundamental band, but it belongs to *ν*_*3*_ rather than *ν*_*14*_. We assigned the band at 1422.3 cm^−1^ in our FFT spectrogram to *ν*_*14*_ through the dual-extreme diagram and normal coordinate relative displacement analyses in the previous subsection. There are two experimental bands in the vicinity of 1400 cm^−1^ in Figs. 6 (a) to (d). Mair and Hornig simply explained the phenomenon as the split of the degenerated combination band *ν*_*16*_ + *ν*_*5*_ in crystal field^42^. Generally speaking, the splitting of the binary combination band in crystal field will result in two bands of similar intensity, and the spacing between the two bands will vary with temperature (see the *ν*_*11*_ + *ν*_*10*_ and *ν*_*17*_ + *ν*_*4*_ combination bands in Mair and Hornig’s work)^42^. However, as shown in Fig. 7, when we carefully examined the relevant original experimental data, we found that when temperature drops, there is an interesting peak intensity reversal between the two experimental bands at about 1400 cm^−1^^42,55,56^. The combination band results from the nonlinear coupling between different vibration modes, and its intensity will weaken as the temperature decreases until it eventually disappears^5,52^. At very low temperature close to 0 K, only the fundamental bands are left. As can be seen from Fig. 7, the behavior of the first and the second bands is completely consistent with the above description of combination band and fundamental band, respectively. Therefore, these two experimental bands are reasonably assigned to *ν*_*16*_ + *ν*_*5*_ and *ν*_*14*_ respectively in this study. As can be seen from Fig. 6, the frequency of the currently assigned experimental *ν*_*14*_ band is also the closest to our theoretical calculation. Our FFT spectrogram in Fig. 3 shows that *ν*_*16*_ and *ν*_*14*_ are respectively the strongest and weakest bands in the molecular vibration of benzene. The intensity of *ν*_*16*_ is approximately 300 times that of *ν*_*14*_. This should be the reason why only *ν*_*16*_ + *ν*_*5*_ was observed in the vicinity of 1400 cm^−1^ in all liquid benzene experiments at room temperature (Supplementary Fig. 4)^34,42,53,60^, and the two-band structure of *ν*_*16*_ + *ν*_*5*_ and *ν*_*14*_ needs to appear at a lower temperature. This double-band structure is similar to the splitting of the degenerated combination band in crystal field, which makes it extremely difficult to be distinguished without the help of our theoretical spectrogram. There is no Fermi resonance between *ν*_*16*_ + *ν*_*5*_ and *ν*_*14*_ bands because of their different symmetry. As a result, their spacing is almost constant as the temperature changes.

The FPMD method used in present research had been successfully applied to the theoretical calculation of vibrational spectra of water, methane, ethylene and cyclobutadiene^3^. We found that the stretching strength of the C–H bond was overestimated and the bending strength of the C–H bond was underestimated for all these molecules by using the same DFT parameter settings as in this study^3^. As shown in Supplementary Table 1, the overestimation of bond stretching strength and the underestimation of bond bending strength also exist for the C–H and C–C bonds of benzene. Both *ν*_*14*_ and *ν*_*18*_ modes can be classified as the C–C stretching vibration. As can be seem from Fig. 6, the frequency of both modes is overestimated compared to the experiment. All of the *ν*_*3*_, *ν*_*9*_, *ν*_*15*_ and *ν*_*19*_ modes involve the C–H bending vibration, therefore their frequencies are all underestimated.

On the basis of the above studies, our results for the vibrational reassignment and band combination analyses of *ν*_*3*_ and *ν*_*14*_ in the well-known experimental IR spectrum of Mair and Hornig are shown in Table 1. According to Angus et al.^33^, if the vibrations of either of two combining bands were allowed in the IR spectrum, all the vibrations of the combination bands will be allowed. It is seen from Table 1 that with the new assignments of *ν*_*3*_ and *ν*_*14*_, the IR bands at 1829 cm^−1^ and 2588 cm^−1^ can be respectively reassigned to* ν*_*14*_ + *ν*_*16*_ and *ν*_*14*_ + *ν*_*9*_, the error of both is − 2 cm^−1^. The band at 2347 cm^−1^ and 2907 cm^−1^ are reassigned to *ν*_*3*_ + *ν*_*18*_ and *ν*_3_ + *ν*_8_ with an error of only − 1 cm^−1^. Considering the anharmonic factor in the formation of the combination band, a little negative absolute error is more reasonable than a positive one. Among these four combination bands, *ν*_*14*_ + *ν*_*9*_ and *ν*_*3*_ + *ν*_*18*_ are typical IR-activated combinations, while *ν*_*14*_ + *ν*_*16*_ and *ν*_3_ + *ν*_8_ are typical Raman-activated combinations in the spectra of gaseous benzene. Due to the relatively loose selection rules, the forbidden bands of the gaseous phase have been observed in the condensed phase from time to time^35,42^.

The Teller–Redlich isotopic product rule^33,40^ is a powerful tool for vibrational assignment verification of isotopic compounds. The *b*_*1g*_ frequencies of 1:4-dideuterobenzene are related to the four *e*_*2g*_ and the *a*_*2g*_ frequencies of benzene by the following product equation^34^:

4 $$\frac{{\prod a_{2g} ,e_{2g} \left( {{\text{C}}_{6} {\text{H}}_{6} } \right)}}{{\prod b_{1g} \left( {1,4 - {\text{C}}_{6} {\text{H}}_{4} {\text{D}}_{2} } \right)}} = 1.367\quad \left( {\text{harmonic value}} \right).$$

Using Brodersen and Langseth's experimental data on C_6_H_6_ and C_6_H_4_D_2_^36^, the calculated product ratios on the left hand of Eq. (4) with our reassigned *ν*_*3*_ (1312 cm^−1^), the recommended values by Mair and Hornig (1340 cm^−1^)^42^ and Brodersen and Langseth (1350 cm^−1^)^36^ of the benzene *a*_*2g*_ vibration are: 1.346, 1.375 and 1.385, respectively. On account of the anharmonic factor, the reasonable value of this ratio should be slightly less than the harmonic value^33^, which provides additional support for our *ν*_*3*_ reassignment. For the *b*_*2u*_ vibration, there is^34,40^:

5 $$\frac{{\prod b_{2u} \left( {{\text{C}}_{6} {\text{H}}_{6} } \right)}}{{\prod b_{2u} \left( {{\text{C}}_{6} {\text{D}}_{6} } \right)}} = 1.414\quad \left( {\text{harmonic value}} \right).$$

The *ν*_*15*_ fundamentals of benzene^42^ and hexadeuterobenzene^12^ had been definitively assigned to 1147 cm^−1^ and 824 cm^−1^, respectively. With Eq. (5) and our reassigned *ν*_*14*_ (1416 cm^−1^), we estimate that the *ν*_*14*_ of C_6_D_6_ should be around 1390 cm^−1^, which is very close to that of C_6_H_6_. This result is consistent with the prediction of Herzfeld et al.that the frequency difference between the two *ν*_*14*_ modes of C_6_H_6_ and C_6_D_6_ should be small, because they both arise from the stretching vibration of benzene’s skeleton carbons^34^. In fact, a weak sharp band was observed at 1393 cm^−1^ in the IR spectrum of liquid C_6_D_6_ as early as 1936 by Bailey et al^61^., and a sharp band with medium intensity at 1390 cm^−1^ was also observed in Miller's experimental IR spectrum of liquid C_6_D_6_^62^. Miller, however, attempted to assign this band to one of the two combination bands *ν*_*6*_ + *ν*_*18*_ and *ν*_*4*_ + *ν*_*17*_. The reason why *ν*_*14*_ band can be more easily observed in liquid C_6_D_6_ should be that the *ν*_*14*_ and the *ν*_*16*_ + *ν*_*5*_, which are entangled together in C_6_H_6_, are very far apart from each other in C_6_D_6_^62^.

## Methods

### Modeling and Simulation Details

To build the structure model for FPMD simulation, the benzene molecule is placed in the center of a vacuum cubic box. The edge of the box is set to 20 Å. Periodical boundary condition is used for solving the Kohn–Sham equation within the framework of density-functional theory. All the first-principles ground-state total energy calculations and FPMD simulations are carried out using the CP2K code^63,64^. The quickstep algorithm^65^ by Gaussian and plane waves formalism, using a dual basis of atom centered Gaussian orbitals and plane waves for regular grids, is adopted for solving DFT Kohn–Sham equations. FPMD simulation is performed using the Quickstep module of CP2K in the scheme of Born–Oppenheimer molecular dynamics (BOMD). The double-zeta valence polarized Gaussian basis sets are chosen for the C and H elements of benzene. These basis sets are optimized for the Goedecker–Teter–Hutter pseudopotentials^66^ and the exchange–correlation functional in the local density approximation (LDA) with Teter–Pade parametrization. 280Ry cutoff for the finest grid level of plane waves is used for GTH pseudopotentials. Orbital transformation methods by conjugate gradient (CG) and direct inversion in the iterative subspace (DIIS) are adopted for wave function calculation in the molecular geometry optimization and MD simulation, respectively^65^. The optimized bond lengths between C–C and C–H atoms of benzene are 1.400 Å and 1.097 Å respectively, which are in good accordance with the experimental measurement, 1.397 Å and 1.084 Å^67^. The microcanonical ensemble (NVE ensemble), in which the total energy, particle number and volume of the system remain unchanged during FPMD simulation, is adopted in this study. NVE FPMD simulation is set up from the optimized ground-state configuration by slightly displacing the atoms from their equilibrium position according to the initial temperature. No any atomic velocity or temperature rescaling is performed throughout the simulation. In this study, the MD timestep was set to 0.1 fs, a total of 10 million steps of molecular dynamics simulations were conducted, and the total energy of the simulation system remained constant throughout. The accuracy and rationality of the above realization in the FPMD calculation of hydrocarbon molecular vibration frequencies have been verified in our previous research^3^.

### FPMD trajectory analyses

Eckart frame algorithm is used for noise reduction and vibrational spectrogram calculation from the raw data of FPMD simulation^3^. Using the scientific calculation packages of Numpy and SciPy, the computer codes of frequency calculation, vibrational assignment and vibrational mode analysis are written in Python language. The animation demonstration of atomic trajectory is realized by Visual Python programming.

## Supplementary information

Supplementary Information 1.

Supplementary Information 2.

## Data Availability

All relevant data are available upon request from the authors.

## Code availability

All relevant codes are available upon request from the authors.
