# Supplementary material for: Intrinsic molecular vibration and rigorous vibrational assignment of benzene by first-principles molecular dynamics
Source: Sci Rep. 2020 Oct 21;10:17875. doi: 10.1038/s41598-020-74872-6 (PMC7578012; doi:10.1038/s41598-020-74872-6)
Supplement: Supplementary file 2 — Supplementary Information 2. [file 41598_2020_74872_MOESM2_ESM.pdf]

Supplementary Information

Intrinsic molecular vibration and rigorous vibrational assignment of benzene by first-principles molecular dynamics

Shaoqing Wang

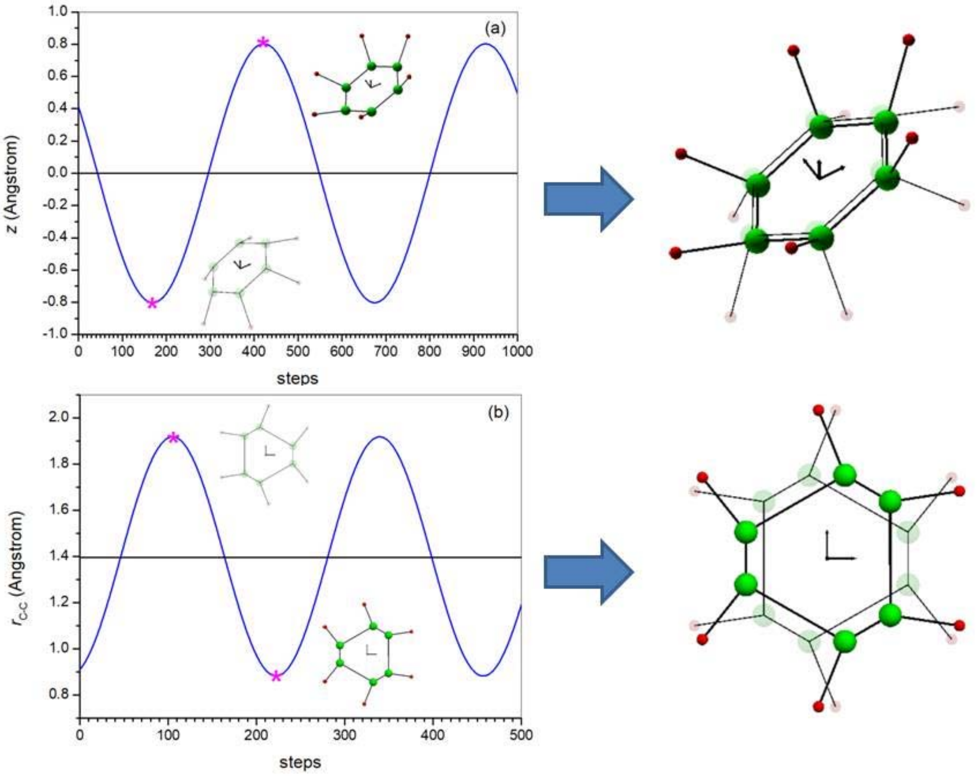

Supplementary Figure 1: The principle of dual-extreme diagram. (a) The dual-extreme diagram constructed based on the trajectory of the *z*-coordinate of a hydrogen atom in benzene with a frequency of 660.7 cm^{-1}. (b) The dual-extreme diagram constructed based on the trajectory of the C-C bond-length of benzene with a frequency of 1422.3 cm^{-1}. The Cartesian coordinate system in dual-extreme diagrams is defined with *z*-axis vertical to the carbon-ring, the *x*- and *y*-axes pass through the midpoint of the two opposite sides and the two diagonal vertices of the carbon hexagon of benzene molecule, respectively.

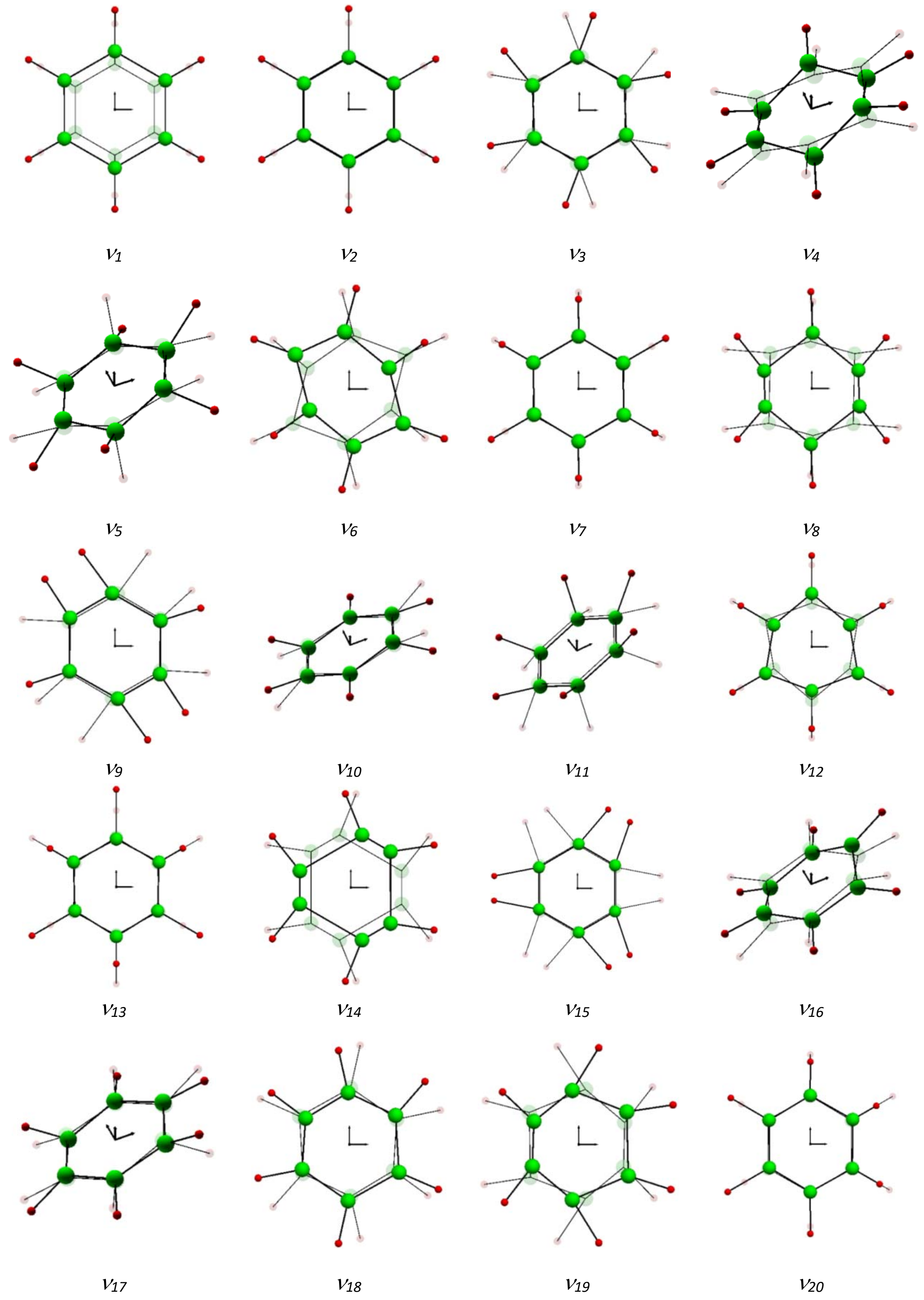

Supplementary Figure 2: The dual-extreme diagrams for the twenty vibrational modes of benzene determined by the frequency-domain filtering IFFT algorithm. The real and virtual diagrams are the two extreme configurations in a vibrational period.

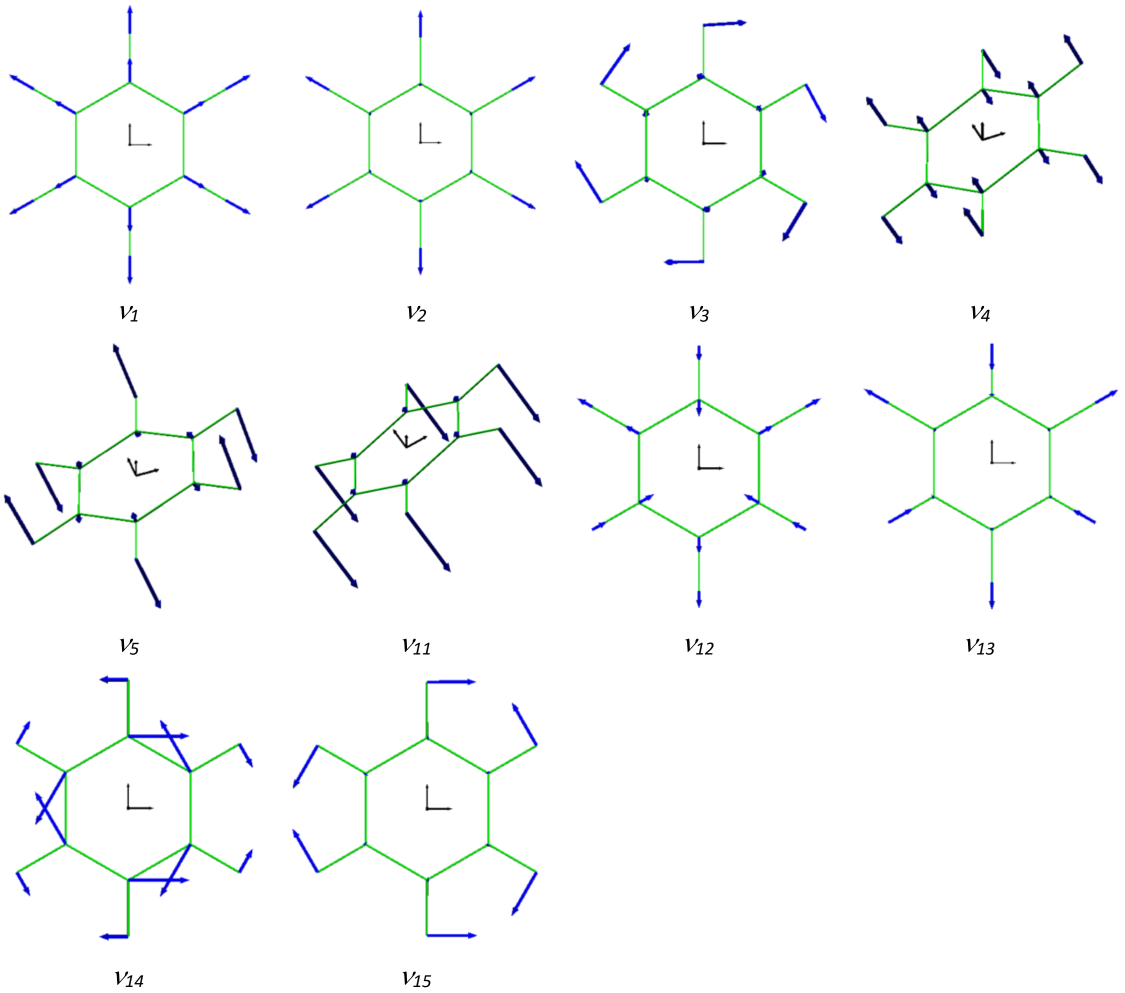

(a) Non-degenerated modes

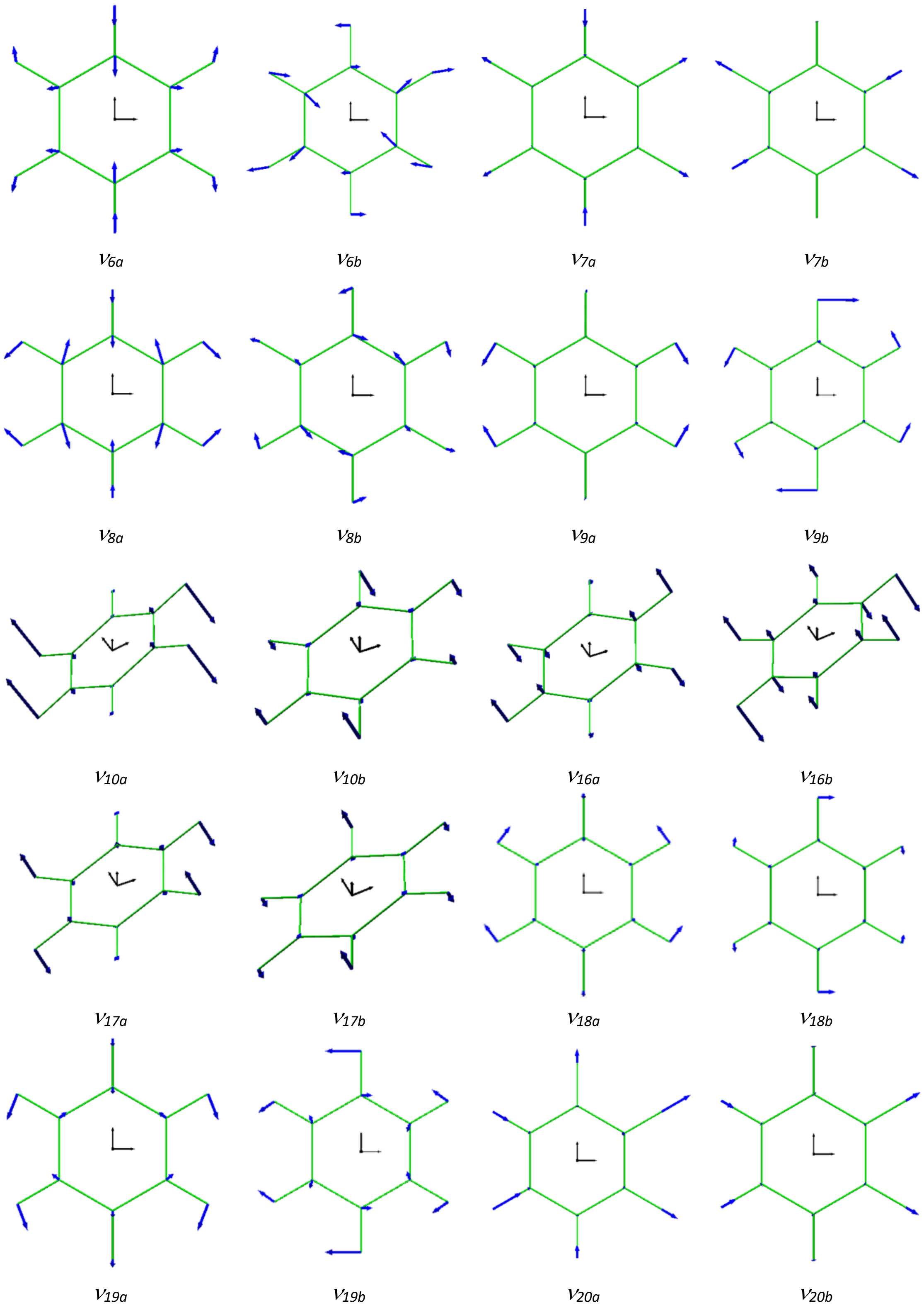

(b) Degenerate modes

Supplementary Figure 3: Normal coordinate relative displacements for benzene's twenty vibrational modes based on the frequency-specified atomic trajectories generated by the frequency-domain filtering IFFT algorithm.

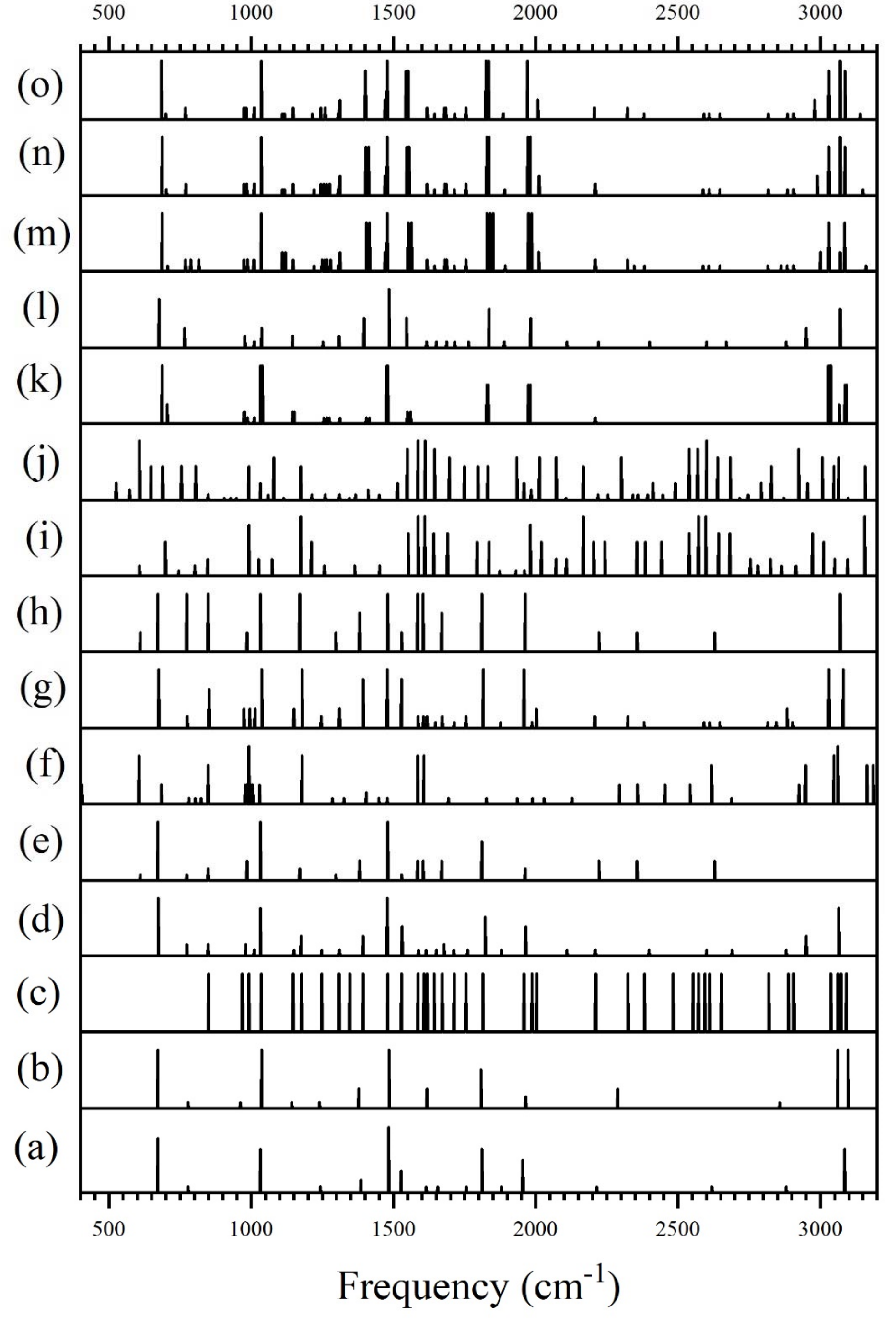

Supplementary Figure 4: Vibrational spectra of benzene by experiments. (a) and (b) gaseous state,[7,12] (c)-(h) liquid state,[7,8,12-14] (i)-(o) solid state.[8,12,15,16]

Supplementary Table 1: Comparisons of the calculated and experimental fundamental frequencies of benzene (unit in cm^{-1}). The modes are named in Wilson numbering system. The experimental data in brackets are estimated and the Mair's experimental data is for crystalline benzene at 103K.

Mode	Sym.	Mode description	Theoretical calculation						Experimental measurement					
			**Present**	Palafox[1]	Yagi[2]	Handy[3]	Martin[4]	Gardner[5]	Pitzer[6]	Ingold[7]	Mair[8]	Brodersen[9]	Varsanyi[10]	NIST[11]
			**FPMD***	SVWN*	cc-VSCF*	MP2*	CCSD(T)*	B3LYP*						
ν_1	a_{1g}	C-C stretching	**1016.7**	1025	1003.0	1015	1004.0	1013	992.5	991.6	990	993	993	992
ν_2	a_{1g}	C-H stretching	**3128.0**	3143	3096.3	3240	3202.7	3201	3061.5	3061.9	3060	3073	3073	3062
ν_3	a_{2g}	C-H bending	**1301.4**	1312	1346.1	1367	1378.6	1354	(1298)	(1326)	(1340)	(1350)	(1350)	(1326)
ν_4	b_{2g}	C-C bending	**700.5**	708	696.8	413	677.3	729	(685)	(703)	(703)	(707)	(707)	(703)
ν_5	b_{2g}	C-H, C-C bending	**971.2**	977	1013.0	903	972.3	1016	(1016)	(985)	(995)	(990)	(990)	(995)
ν_6	e_{2g}	C-C bending	**591.7**	601	602.9	610	606.9	616	606.4	605.6	606	606	606	606
ν_7	e_{2g}	C-H stretching	**3101.3**	3120	3075.1	3215	3174.4	3176	3048.3	3046.8	3040	3056	3056	3047
ν_8	e_{2g}	C-C stretching	**1631.9**	1644	1620.0	1645	1636.3	1634	(1595)	(1596)	(1596)	1599	1599	(1596)
ν_9	e_{2g}	C-H bending	**1145.8**	1161	1182.7	1199	1189.6	1188	1177.9	1178.0	1174	1178	1178	1178
ν_{10}	e_{1g}	C-H, C-C bending	**828.0**	833	878.4	842	858.5	857	849.7	848.9	854	846	846	849
ν_{11}	a_{2u}	C-H bending	**660.7**	665	728.2	678	685.6	681	671	671	687	673	673	673
ν_{12}	b_{1u}	C-C bending	**973.1**	981	1002.0	1009	1011.7	1005	(1010)	(1010)	(1010)	(1010)	(1010)	(1010)
ν_{13}	b_{1u}	C-H stretching	**3092.0**	3111	3082.6	3204	3163.0	3168	(3060)	(3060)	3069	(3057)	(3057)	(3068)
ν_{14}	b_{2u}	C-C stretching	**1422.3**	1423	1444.9	1451	1326.5	1351	(1693)	(1648)	1312	1309	1309	1310
ν_{15}	b_{2u}	C-H bending	**1117.8**	1135	1161.4	1173	1158.1	1164	(1170)	(1110)	1147	1146	1146	1150
ν_{16}	e_{2u}	C-C bending	**392.9**	399	408.1	389	401.9	413	(400)	(405)	(415)	(398)	(404)	(410)
ν_{17}	e_{2u}	C-H bending	**945.4**	935	981.5	908	962.6	982	(985)	(970)	975	(967)	(967)	975
ν_{18}	e_{1u}	C-C stretching	**1042.1**	1050	1053.8	1063	1053.7	1056	1035	1037	1036	1037	1037	1038
ν_{19}	e_{1u}	C-H bending	**1460.8**	1481	1484.8	1509	1505.4	1493	1485	1485	1478	1482	1482	1486
ν_{20}	e_{1u}	C-H stretching	**3117.2**	3134	3090.2	3230	3192.0	3191	3080	3080	3063	3064	3064	3063

*Method used in the calculation.

Supplementary References

1. Palafox, M. A. Scaling factors for the prediction of vibrational spectra. I. Benzene molecule. *Inter. J. Quant. Chem.* **77**, 661-684 (2000).
2. Yagi, K., Hirao, K., Taketsugu, T., Schmidt, M. W. & Gordon, M. S. Ab initio vibrational state calculations with a quartic force field: Applications to H_2CO, C_2H_4, CH_3OH, CH_3CCH, and C_6H_6. *J. Chem. Phys.* **121**, 1383-1389 (2004).
3. Handy, N. C. et al. The harmonic frequencies of benzene. *Chem. Phys. Lett.* **197**, 506-515 (1992).
4. Martin, J. M. L., Taylor, P. R. & Lee, T. J. The harmonic frequencies of benzene. A case for atomic natural orbital basis sets. *Chem. Phys. Lett.* **275**, 414-422 (1997).
5. Gardner, A. M. & Wright, T. G. Consistent assignment of the vibrations of monosubstituted benzene. *J. Chem. Phys.* **135**, 114305-17 (2011).
6. Pitzer, K. S. & Scott, D. W. The thermodynamics and Molecular structure of benzene and its methyl derivatives. *J. Am. Chem. Soc.* **65**, 803-829 (1943).
7. Herzfeld, N., Ingold, C. K. & Poole, H. G. Structure of benzene. Part XXI. The inactive fundamental frequencies of benzene, hexadeuterobenzene, and the partly deuterated benzenes. *J. Chem. Soc. (London)* 316-333 (1946).
8. Mair, D. & Hornig, D. F. The vibrational spectra of molecules and complex ions in crystals. II. Benzene. *J. Phys. Chem.* **17**, 1236-1247 (1949).
9. Brodersen, S. & Langseth, A. *Mat. Fys. Skr. Dan. Vid. Selsk.* **1**, 1 (1956).
10. Varsanyi, G. Vibrational spectra of benzene derivatives. (Academic Press, 1969).
11. NIST Chemistry WebBook, https://webbook.nist.gov/chemistry/.
12. Halford, R. S. & Schaeffer, O. A. Motions of molecules in condensed system: II. The infra-red spectra for benzene solid, liquid, and vapor in the range from 3 to 16.7μ. *J. Chem. Phys.* **14**, 141-149 (1946).
13. Brodersen, S., Christoffersen, J., Bak, B. & Nielsen, J. T. The infrared spectrum of mono-^{13}C-substituted benzene. *Spectrochim. Acta* **21**, 2077-2084 (1965).
14. Bailey, C. R., Hale, J. B., Ingold, C. K. & Thompson, J. W. Structure of benzene. Part IV. Infra-red absorption spectra of benzene and hexadeuterobenzene both as

vapour and as liquid. *J. Chem. Soc. (London)* 931-941 (1936).

15. Swenson, C. A. & Person, W. B. Infrared studies of crystal benzene. II. Relative intensities. *J. Chem. Phys.* **33**, 56-64 (1960).
16. Spangler, J. D. & Kilmer, N. G. Electronic spectra of benzene in cyclohexane at 77 K. *J. Chem. Phys.* **48**, 698-714 (1968).

Description of Additional Supplementary Files

File Name: 1016.7_nu1.mov
Description: Animation file of benzene molecule vibrating at the frequency of 1016.7 cm^{-1} by the frequency-domain filtering algorithm. This fundamental frequency is assigned to mode ν_1 by the dual-extreme diagram and the normal coordinate relative displacement analyses.

File Name: 3128.0_nu2.mov
Description: Animation file of benzene molecule vibrating at the frequency of 3128.0 cm^{-1} by the frequency-domain filtering algorithm. This fundamental frequency is assigned to mode ν_2 by the dual-extreme diagram and the normal coordinate relative displacement analyses.

File Name: 1301.4_nu3.mov
Description: Animation file of benzene molecule vibrating at the frequency of 1301.4 cm^{-1} by the frequency-domain filtering algorithm. This fundamental frequency is assigned to mode ν_3 by the dual-extreme diagram and the normal coordinate relative displacement analyses.

File Name: 700.5_nu4.mov
Description: Animation file of benzene molecule vibrating at the frequency of 700.5 cm^{-1} by the frequency-domain filtering algorithm. This fundamental frequency is assigned to mode ν_4 by the dual-extreme diagram and the normal coordinate relative displacement analyses.

File Name: 971.2_nu5.mov
Description: Animation file of benzene molecule vibrating at the frequency of 971.2 cm^{-1} by the frequency-domain filtering algorithm. This fundamental frequency is assigned to mode ν_5 by the dual-extreme diagram and the normal coordinate relative displacement analyses.

File Name: 591.7_nu6.mov
Description: Animation file of benzene molecule vibrating at the frequency of 591.7 cm^{-1} by the frequency-domain filtering algorithm. This fundamental frequency is assigned to mode ν_6 by the dual-extreme diagram and the normal coordinate relative displacement analyses.

File Name: 3101.3_nu7.mov
Description: Animation file of benzene molecule vibrating at the frequency of 3101.3 cm^{-1} by the frequency-domain filtering algorithm. This fundamental frequency is assigned to mode ν_7 by the dual-extreme diagram and the normal coordinate relative

displacement analyses.

File Name: 1631.9_nu8.mov
Description: Animation file of benzene molecule vibrating at the frequency of 1631.9 cm^{-1} by the frequency-domain filtering algorithm. This fundamental frequency is assigned to mode ν_8 by the dual-extreme diagram and the normal coordinate relative displacement analyses.

File Name: 1145.8_nu9.mov
Description: Animation file of benzene molecule vibrating at the frequency of 1145.8 cm^{-1} by the frequency-domain filtering algorithm. This fundamental frequency is assigned to mode ν_9 by the dual-extreme diagram and the normal coordinate relative displacement analyses.

File Name: 828.0_nu10.mov
Description: Animation file of benzene molecule vibrating at the frequency of 828.0 cm^{-1} by the frequency-domain filtering algorithm. This fundamental frequency is assigned to mode ν_{10} by the dual-extreme diagram and the normal coordinate relative displacement analyses.

File Name: 660.7_nu11.mov
Description: Animation file of benzene molecule vibrating at the frequency of 660.7 cm^{-1} by the frequency-domain filtering algorithm. This fundamental frequency is assigned to mode ν_{11} by the dual-extreme diagram and the normal coordinate relative displacement analyses.

File Name: 973.1_nu12.mov
Description: Animation file of benzene molecule vibrating at the frequency of 973.1 cm^{-1} by the frequency-domain filtering algorithm. This fundamental frequency is assigned to mode ν_{12} by the dual-extreme diagram and the normal coordinate relative displacement analyses.

File Name: 3092.0_nu13.mov
Description: Animation file of benzene molecule vibrating at the frequency of 3092.0 cm^{-1} by the frequency-domain filtering algorithm. This fundamental frequency is assigned to mode ν_{13} by the dual-extreme diagram and the normal coordinate relative displacement analyses.

File Name: 1422.3_nu14.mov
Description: Animation file of benzene molecule vibrating at the frequency of 1422.3 cm^{-1} by the frequency-domain filtering algorithm. This fundamental frequency is assigned to mode ν_{14} by the dual-extreme diagram and the normal coordinate relative displacement analyses.

File Name: 1117.8_nu15.mov
Description: Animation file of benzene molecule vibrating at the frequency of 1117.8 cm^{-1} by the frequency-domain filtering algorithm. This fundamental frequency is assigned to mode ν_{15} by the dual-extreme diagram and the normal coordinate relative displacement analyses.

File Name: 392.9_nu16.mov
Description: Animation file of benzene molecule vibrating at the frequency of 392.9 cm^{-1} by the frequency-domain filtering algorithm. This fundamental frequency is assigned to mode ν_{16} by the dual-extreme diagram and the normal coordinate relative displacement analyses.

File Name: 945.4_nu17.mov
Description: Animation file of benzene molecule vibrating at the frequency of 945.4 cm^{-1} by the frequency-domain filtering algorithm. This fundamental frequency is assigned to mode ν_{17} by the dual-extreme diagram and the normal coordinate relative displacement analyses.

File Name: 1042.1_nu18.mov
Description: Animation file of benzene molecule vibrating at the frequency of 1042.1 cm^{-1} by the frequency-domain filtering algorithm. This fundamental frequency is assigned to mode ν_{18} by the dual-extreme diagram and the normal coordinate relative displacement analyses.

File Name: 1460.8_nu19.mov
Description: Animation file of benzene molecule vibrating at the frequency of 1460.8 cm^{-1} by the frequency-domain filtering algorithm. This fundamental frequency is assigned to mode ν_{19} by the dual-extreme diagram and the normal coordinate relative displacement analyses.

File Name: 3117.2_nu20.mov
Description: Animation file of benzene molecule vibrating at the frequency of 3117.2 cm^{-1} by the frequency-domain filtering algorithm. This fundamental frequency is assigned to mode ν_{20} by the dual-extreme diagram and the normal coordinate relative displacement analyses.

File Name: 1560.6_H2O.mov
Description: Animation file of water monomer vibrating at the frequency of 1560.6 cm^{-1} by the frequency-domain filtering algorithm. This fundamental frequency is assigned to the bending mode of a_1 symmetry by the dual-extreme diagram analysis.

File Name: 3711.1_H2O.mov
Description: Animation file of water monomer vibrating at the frequency of 3711.1 cm^{-1} by the frequency-domain filtering algorithm. This fundamental frequency is

assigned to the symmetric stretching mode of a_1 symmetry by the dual-extreme diagram analysis.

File Name: 3819.3_H2O.mov
Description: Animation file of water monomer vibrating at the frequency of 3819.3 cm^{-1} by the frequency-domain filtering algorithm. This fundamental frequency is assigned to the asymmetric stretching mode of a_1 symmetry by the dual-extreme diagram analysis.
